# Carbapenem-Resistant *Klebsiella pneumoniae* in Romania and South-Eastern Europe: A Multilingual Narrative Review of Pandemic-Era Trajectories, 2020–2025

**DOI:** 10.3390/pathogens15101004

**Published:** 2026-09-22

**Authors:** Roxana-Carmen Cernat, Oana-Elena Ioniţă, Maria-Elena Vodarici, Nicola-Maria Militaru, Rareș-Alexandru Constantin, Nela-Daniela Efrim, Elena Mocanu, Edmond Puca, Daniela Piţigoi, Valeriu Gheorghiţă, Irina-Magdalena Dumitru

**Affiliations:** 1 Department of Infectious Diseases and Epidemiology, Faculty of Medicine, Ovidius University of Constanta, 1 University Alley, 900470 Constanta, Romania; roxana.cernat@365.univ-ovidius.ro (R.-C.C.); irina.dumitru@365.univ-ovidius.ro (I.-M.D.); 2 Clinical Infectious Diseases Hospital, Ferdinand Blvd. No. 100, 900709 Constanța, Romania; nicolamilitaru@gmail.com (N.-M.M.); daniela.efrim@gmail.com (N.-D.E.); 3 Doctoral School, Faculty of Medicine, Ovidius University of Constanta, 1 University Alley, 900470 Constanta, Romania; 4 County Clinical Emergency Hospital “St. Apostle Andrew”, Tomis Blvd. No. 145, 900591 Constanța, Romania; rctin96@gmail.com; 5 Department of Public Health and Management, Faculty of Medicine, Ovidius University of Constanta, 1 University Alley, 900470 Constanta, Romania; drmocanu@yahoo.com; 6 Service of Infectious Diseases, University Hospital Center “Mother Teresa”, 1007 Tirane, Albania; edmond_puca@yahoo.com; 7 Department of Epidemiology I, University of Medicine and Pharmacy “Carol Davila”, Bd. Eroii Sanitari 8, Sector 5, 050474 Bucharest, Romania; daniela.pitigoi@umfcd.ro; 8 National Institute of Infectious Diseases “Prof. Dr. Matei Balș”, 1 Dr. Calistrat Grozovici Street, District 2, 021105 Bucharest, Romania; 9 Faculty of Medicine, University of Medicine and Pharmacy “Carol Davila”, Bd. Eroii Sanitari 8, Sector 5, 050474 Bucharest, Romania; valeriu.gheorghita@umfcd.ro; 10 “Prof. Dr. Agrippa Ionescu” Emergency Clinical Hospital, 077015 Bucharest, Romania; 11 Academy of Romanian Scientists, 3 Ilfov Street, 050044 Bucharest, Romania

**Keywords:** *Klebsiella pneumoniae*, carbapenem resistance, healthcare-associated infections, Romania, South-Eastern Europe, Balkans, COVID-19, molecular epidemiology, antimicrobial surveillance, NDM, OXA-48

## Abstract

Carbapenem-resistant *Klebsiella pneumoniae* (CRKP) is the pathogen driving the deterioration of the European antimicrobial resistance landscape, and the burden falls disproportionately on Romania and South-Eastern Europe. The objective of this review was to synthesise evidence published between 2020 and 2025 on the epidemiology, molecular characteristics, clinical impact, and pandemic-era trajectory of CRKP healthcare-associated infections across South-Eastern Europe, including Romania. This retrospective, qualitative narrative review combined the international literature with a deliberately multilingual search of national-language sources in Romanian, Bulgarian, Greek, Croatian, Slovenian, and Serbian, including congress abstract books, doctoral theses, national reference-laboratory reports, and agency surveillance series that are absent from PubMed. Three findings emerge. First, the region is not epidemiologically uniform: estimated incidence of carbapenem-resistant *K. pneumoniae* bloodstream infection in 2024 ranged from 0.62 per 100,000 population in Slovenia to 20.31 in Romania, against a European Union/European Economic Area (EU/EEA) figure of 3.46, and the steepest relative rise between 2019 and 2024 was recorded in Bulgaria (+374.1%). Second, a distinctive molecular signature has consolidated in the northern half of the region: co-production of a metallo-β-lactamase with an OXA-48-like enzyme (OXA-48, a class D, oxacillinase-type carbapenemase), reported in 44.4–61.2% of characterised Romanian isolates across independent centres, which removes ceftazidime-avibactam from the therapeutic repertoire and leaves aztreonam-avibactam and cefiderocol as the principal remaining options. Third, the pandemic did not produce a single regional signal but a consistent sequence—a transient interruption of upward trends during 2020, a marked acceleration from 2021, and persistence of the elevated level after 2022—with national-language surveillance from Croatia and Slovenia showing that the direction of the apparent pandemic effect depends on whether incidence or resistance proportion is measured. The national-language literature adds granularity unavailable in English, but the evidence base remains structurally uneven: Albania, Kosovo, and North Macedonia contribute almost no primary data, and no country in the region has published a CRKP-attributable mortality estimate for the review period. CRKP in Romania and South-Eastern Europe now maps onto three molecular zones—OXA-48 predominance in the Western Balkans and Croatia, a KPC-to-metallo-β-lactamase transition in Greece, and metallo-β-lactamase plus OXA-48-like co-production in Romania and increasingly Bulgaria—that require different empirical therapy; the COVID-19 pandemic did not create this burden but accelerated and entrenched it, and closing the region’s mortality-data and Western Balkan surveillance gaps should be a priority for future research.

## 1. Introduction

Healthcare-associated infections (HAIs) remain the most frequent adverse event in hospital care. In the most recent European point prevalence survey, conducted in 2022–2023 across 1332 hospitals and 309,504 patients in 28 European Union/European Economic Area (EU/EEA) countries and three Western Balkan countries, the crude prevalence of patients with at least one HAI was 7.1%, with a country range of 3.1–13.8% [1]. Within that burden, *Klebsiella pneumoniae* occupies a position of particular concern: in the same survey, 25.1% of *K. pneumoniae* isolates from HAIs were carbapenem-resistant, the highest proportion of any *Enterobacterales* species, and 58.1% were resistant to third-generation cephalosporins [1]. The World Health Organization retained carbapenem-resistant *Enterobacterales* in the critical priority category of its 2024 Bacterial Priority Pathogens List [2], and the Global Burden of Disease analysis of antimicrobial resistance found that carbapenem resistance had increased more than resistance to any other antibiotic class among Gram-negative organisms, with associated deaths rising from approximately 619,000 in 1990 to 1.03 million in 2021 [3].

At European level the aggregate figures conceal an east–west gradient of a magnitude that makes the European mean almost uninterpretable as a description of any individual country. The EU/EEA population-weighted mean percentage of invasive *K. pneumoniae* isolates resistant to carbapenems rose from 10.4% in 2019 to 13.3% in 2023, but the country range in 2023 spanned 0.0–69.7% [4]. Expressed as estimated incidence of carbapenem-resistant *K. pneumoniae* bloodstream infection, the EU/EEA figure for 2024 was 3.46 per 100,000 population, against a country range of 0.02–20.31 [5]. Romania sits at the upper end of that range, and its immediate neighbours occupy much of the rest of the upper half.

This regional concentration has been recognised repeatedly but analysed only partially. The Balkan carbapenem-resistant CRKP review of Chatzidimitriou and colleagues covered nine countries over more than two decades and established the broad clonal architecture of the region [6]; Brkić and Ćirković mapped the surveillance void in the Western Balkans, where several countries fall outside the European Antimicrobial Resistance Surveillance Network (EARS-Net) and are captured only partially by the WHO Central Asian and European Surveillance of Antimicrobial Resistance network (CAESAR) [7]; and Piotrowski and colleagues conducted a systematic review of carbapenem-resistant *Enterobacterales* and *Pseudomonas aeruginosa* across ten Eastern European countries, concluding that real-world treatment data from the region are conspicuously scarce [8]. Complementing these regional analyses, our group has separately synthesised the global epidemiology, resistance mechanisms, and therapeutic strategies of CRKP with a dedicated focus on Romania and Eastern Europe [9]; the present review narrows that lens specifically to South-Eastern Europe, extends the search to six additional national languages, and concentrates on the pandemic-era trajectory (2020–2025). Concretely, ref. [9] addressed CRKP as a global entity, synthesising worldwide resistance mechanisms and therapeutic strategies with Romania and Eastern Europe presented as one illustrative regional case among many, and did not undertake a systematic national-language search; the present review inverts that scope, treating Romania and the countries of South-Eastern Europe as the primary unit of analysis, adding six further national languages to the search, and reorganising the regional evidence explicitly around a pre-pandemic, pandemic, and post-pandemic comparison—a temporal framework not used in the earlier paper. The two publications share no tables, figures, or pooled analyses; the national-language sources retrieved specifically for this review were identified independently of, and in several cases post-date, the search underlying [9]. What none of these syntheses attempted was a systematic interrogation of the national-language literature. That omission matters. Much of the most granular epidemiological information produced in this region—national point-prevalence survey results, reference-laboratory carbapenemase distributions, hospital-level pre- and post-pandemic comparisons—is published in Romanian, Bulgarian, Greek, Croatian, Slovenian, or Serbian, in congress abstract books, doctoral theses, agency reports, and national journals that are not indexed in PubMed, Embase, or Web of Science. A review restricted to English-language sources therefore systematically under-represents precisely the countries whose data are most needed.

A second gap concerns time. The COVID-19 pandemic coincided with the steepest documented rise in carbapenem resistance in the region’s recorded history, and a substantial literature now exists on antibiotic overprescribing in COVID-19 patients [10,11], on the collapse of infection prevention and control capacity under caseload pressure [12,13], and on the divergent behaviour of resistance incidence and resistance proportion during the pandemic [14,15]. Yet the region-specific question—what happened to CRKP in Romania and South-Eastern Europe before, during, and after the pandemic, and whether the pandemic-era changes proved transient or permanent—has not been assembled from the available evidence.

The present narrative review addresses both gaps. It synthesises evidence published between 2020 and 2025 on the epidemiology, molecular epidemiology, clinical outcomes, and therapeutic management of CRKP healthcare-associated infections in Romania and South-Eastern Europe, with an explicitly multilingual evidence base, and organises the temporal evidence around a pre-pandemic, pandemic, and post-pandemic comparison wherever the primary data permit that stratification. It is intended as a regional companion to, rather than a repetition of, the global and mechanistic literature. Romania is treated throughout this review as one of the countries of South-Eastern Europe, not as a separate entity; it is named individually in the title and text because it supplies the largest share of the regional evidence base and anchors the upper end of the resistance gradient described in Section 3, a distinction of evidentiary weight rather than of geographic classification.

## 2. Literature Search Strategy

The search strategy described in this section was designed to meet the two objectives set out in Section 1: an explicitly multilingual synthesis of the evidence for Romania and South-Eastern Europe, and a pre-pandemic, pandemic, and post-pandemic stratification of that evidence wherever the primary data permit it.

This is a retrospective, qualitative narrative review of previously published, secondary quantitative and qualitative evidence. It is non-systematic in design: it was not registered to a protocol, study selection was not performed in duplicate, no formal risk-of-bias instrument was applied, and no quantitative meta-analysis was undertaken (see the limitations discussed in Section 8.3). All pooled estimates presented below are reproduced from the published analyses cited and are attributed to their sources.

### 2.1. Eligibility Criteria

Eligibility followed an adapted PICOS (Population, Intervention/Exposure, Comparator, Outcomes, Study design) framework. Population: patients with healthcare-associated infection due to carbapenem-resistant *Klebsiella pneumoniae*, in Romania or another country of South-Eastern Europe (Bulgaria, Greece, Croatia, Slovenia, Serbia, Bosnia and Herzegovina, Montenegro, North Macedonia, Albania, Kosovo). Exposure/comparison: pre-pandemic (before March 2020), pandemic (March 2020–2022), and post-pandemic (2023–2025) periods, compared where the primary data permitted. Outcomes: incidence or prevalence of carbapenem resistance or carbapenemase-mediated resistance, molecular epidemiology (carbapenemase gene, sequence type), clinical outcome (mortality, length of stay, treatment response) or antimicrobial consumption. Study design: surveillance reports, cohort studies, cross-sectional and point-prevalence surveys, outbreak reports, and systematic or narrative reviews providing regional syntheses; case reports of a single patient were excluded unless they documented a novel resistance mechanism not otherwise described in the region. Sources were included irrespective of language, publication venue (peer-reviewed journal, congress abstract, agency report, doctoral thesis) or indexing status, provided the full text or a sufficiently detailed abstract could be retrieved and the country and study period could be established. Publications reporting exclusively on organisms other than *K. pneumoniae*, on countries outside the defined region, or predating January 2020 (except where cited for pre-pandemic baseline context) were excluded, as were sources for which the underlying data could not be retrieved in any form. Title/abstract screening and full-text assessment against these criteria were performed by one reviewer, a limitation made explicit in Section 8.3.

Because the same source can appear in more than one of the four international databases, or in both an international database and a national-language repository, duplicate records were expected and were removed before inclusion; the manual, cross-database and cross-language matching procedure used is described in Section 2.5.

### 2.2. International Databases

PubMed/MEDLINE, Embase, Web of Science Core Collection, and the Cochrane Central Register of Controlled Trials were searched for publications appearing between January 2020 and December 2025, combining terms for the organism (*Klebsiella pneumoniae*, CRKP, carbapenemase-producing *Enterobacterales*), the resistance phenotype and genotype (carbapenem resistance, KPC, NDM, OXA-48, VIM, colistin, *mcr*, *mgrB*), the setting (healthcare-associated infection, nosocomial, intensive care unit), and each country of interest. Reference lists of retrieved reviews were hand-searched. Priority was given to multicentre studies, national and supranational surveillance reports, whole-genome-sequencing-based molecular epidemiology and clinical guidelines; single-centre series were included where they supplied country-level data otherwise unavailable.

### 2.3. National-Language Searching

The distinguishing methodological feature of this review is a deliberate search of sources published in the national languages of the region. Searches were conducted in Romanian, Bulgarian (Cyrillic), Greek, Croatian, Slovenian, and Serbian (Latin and Cyrillic), and in Albanian and Macedonian. Targets included: national journals not indexed in the international databases; congress abstract volumes of the national microbiology and infectious diseases societies; doctoral thesis repositories of the national medical universities; and the publication series of national public health institutes and reference laboratories.

This search yielded material of a kind that is simply absent from the international databases—for example, the annual reports of the Bulgarian National Reference Centre for Healthcare-Associated Infections presented at the national clinical microbiology congress [16], the Croatian national antibiotic susceptibility series issued by the Croatian Academy of Medical Sciences [17,18], the Slovenian EARS-Net national reports of the National Institute of Public Health [19,20], the surveillance report of the Greek National Public Health Organisation and the Greek national action plan [21,22], and the abstract volumes of the Romanian Society of Microbiology, which contain the only Romanian-language pre- versus post-pandemic CRKP comparison identified in this review [23,24]. Greek and Bulgarian doctoral thesis repositories yielded further datasets that exist in no other form [25,26].

Two structural observations about this literature deserve to be recorded, because they bear on how the regional evidence base should be read. First, national-language journal publication on this topic has largely ceased: a PubMed query restricted to Serbian, Croatian or Slovenian as the publication language, combined with *Klebsiella*, carbapenem, antimicrobial resistance or nosocomial infection terms, returned 75 records, every one of them dated 2017 or earlier. The contemporary national-language evidence therefore lives almost entirely in agency reports and congress proceedings rather than in journals. Second, Bulgarian-language journal literature on CRKP is effectively invisible to the international databases, and the Bulgarian national reference laboratory’s own journal blocks automated retrieval; the Bulgarian evidence used here was recovered from national congress abstract books and university repositories.

### 2.4. Methodological Caveats

Three caveats apply throughout and should be borne in mind when interpreting the figures presented below.

First, sources differ in the denominator to which a resistance figure refers. Supranational surveillance reports resistance among invasive (blood and cerebrospinal fluid) isolates, or as estimated bloodstream-infection incidence per 100,000 population; hospital studies typically report resistance among all clinical isolates, or among carbapenem-non-susceptible isolates referred for molecular characterisation. These are not interchangeable, and the difference explains much of the apparent inconsistency between national and single-centre figures. Where both are cited in the same paragraph, the denominator is stated.

Second, molecular characterisation capacity is unevenly distributed across the region. Countries with established genomic surveillance are more likely to have their circulating carbapenemases and clones described; the absence of a genotype from a country’s row in the tables below indicates absence of evidence rather than evidence of absence.

Third, several national-language sources are congress abstracts or agency documents for which complete bibliographic fields, or verification of internal figures against the primary document, could not be established from the retrieved record. These are identified as such in the text and in the tables, and the corresponding figures are presented as reported rather than as independently confirmed.

### 2.5. Search Execution and Verification

Searches were executed in a single coordinated pass completed in December 2025, timed to allow inclusion of sources published through the end of the review period, and were not subsequently re-run. No automated cross-database or cross-language deduplication tool was used, given the heterogeneous indexing of congress abstracts, theses and agency reports; records were instead deduplicated manually by matching institution, cohort, study period and reported figures across sources. An illustrative PubMed search string, adapted per database syntax and repeated with the national-language equivalent of each term for the searches described in Section 2.3, was: (“*Klebsiella pneumoniae*”[Title/Abstract] OR CRKP[Title/Abstract]) AND (carbapenem*[Title/Abstract] OR KPC[Title/Abstract] OR NDM[Title/Abstract] OR “OXA-48”[Title/Abstract] OR VIM[Title/Abstract]) AND (Romania[Title/Abstract] OR Bulgaria[Title/Abstract] OR Greece[Title/Abstract] OR Croatia[Title/Abstract] OR Slovenia[Title/Abstract] OR Serbia[Title/Abstract] OR “Bosnia and Herzegovina”[Title/Abstract] OR Montenegro[Title/Abstract] OR “North Macedonia”[Title/Abstract] OR Albania[Title/Abstract] OR Kosovo[Title/Abstract]). For non-English sources, the quantitative findings reported in this review were extracted and re-verified directly from the original-language text, tables or figures by a team member fluent in that language, rather than from translated abstracts alone, to reduce transcription error.

## 3. The Regional Epidemiological Landscape

This section addresses the first of the two objectives set out in Section 1—whether the region is epidemiologically uniform—using the supranational and national surveillance sources identified in Section 2.

### 3.1. Supranational Surveillance and Its Blind Spot

The single most informative regional dataset is the estimated incidence of carbapenem-resistant *K. pneumoniae* bloodstream infection published by the European Centre for Disease Prevention and Control (ECDC), reproduced in Table 1. Read across the six years it covers, this table makes three points that no single-centre study can make.

The first is the magnitude of the gradient. In 2024 the estimated incidence ranged from 0.62 per 100,000 population in Slovenia to 20.31 in Romania—a thirty-three-fold difference between two countries separated by less than 500 km, both members of the European Union, both reporting through the same surveillance protocol. Romania’s figure is the highest in the EU/EEA and is almost six times the EU/EEA estimate of 3.46 [5].

The second is the direction and steepness of change. Every country in the region for which EARS-Net data exist recorded an increase between 2019 and 2024, but the rates of increase differ markedly. Bulgaria’s incidence rose by 374.1%, from 2.24 to 10.62 per 100,000—the steepest proportional rise of any EU/EEA country and a change that the ECDC risk assessment flags as statistically significant [27]. Croatia rose by 274.2% and Slovenia by 1140%, although the latter figure is derived from a very small base (0.05 to 0.62) and should not be read as indicating a Slovenian problem comparable in absolute terms to Romania’s. Romania itself rose by 185.3%, from an already high baseline.

The third is that Greece behaves differently from the rest of the region. Greek incidence peaked at 23.30 per 100,000 in 2021—the highest single-country-year value in the table—and then fell to 14.89 by 2024, an overall change of only +14.1% across the six years and the only trajectory in the region that is not monotonically upward [5,27]. This pattern, a pandemic-era peak followed by a partial retreat, distinguishes Greece from Romania and Bulgaria, where the pandemic-era rise was followed by consolidation at the higher level rather than retreat.

These divergent trajectories are summarised in Figure 1.

Against these data sits a substantial blind spot. Serbia, Bosnia and Herzegovina, Montenegro, North Macedonia, Albania, and Kosovo are not EARS-Net participants; they report through CAESAR, whose country-level tables are not reproduced in the ECDC annual epidemiological reports and were not retrievable in a form permitting verification for this review [7,28]. The consequence is that for six of the eleven jurisdictions considered here—a population of roughly 17 million—no comparable annual incidence series exists. Brkić and Ćirković have argued that this gap is not a technical artefact but a resource constraint, and that the Western Balkans are consequently absent from the European AMR surveillance map at precisely the point on the east–west gradient where the data would be most informative [7]. The country-level evidence base is summarised in Table 2.

**Table 1:** Estimated incidence of carbapenem-resistant *Klebsiella pneumoniae* bloodstream infections per 100,000 population, EARS-Net countries of South-Eastern Europe, 2019–2024.

Country	2019	2020	2021	2022	2023	2024	Change 2019–2024
Romania	7.12	10.77	13.87	12.12	20.02	20.31	+185.3%
Greece	13.05	14.96	23.30	18.02	21.44	14.89	+14.1%
Bulgaria	2.24	2.19	3.52	3.91	7.75	10.62	+374.1%
Croatia	1.20	1.57	2.87	2.52	4.53	4.49	+274.2%
Slovenia	0.05	0.00	0.14	0.34	0.62	0.62	+1140.0%
EU/EEA	2.15	2.34	2.69	2.62	3.35	3.46	+60.9%
CAESAR countries a	—	—	—	—	—	—	Not reported

a Serbia, Bosnia and Herzegovina, Montenegro, North Macedonia, Albania, and Kosovo. All values reproduced from the ECDC EARS-Net Annual Epidemiological Report for 2024 [5]; percentage changes as printed in that source. Values for Romania, Bulgaria, and Greece carry the ECDC footnote flag for limited or changing coverage in several years, and should be interpreted accordingly. Slovenia’s percentage change is derived from a very small base and does not indicate an absolute burden comparable to that of the other countries listed. The six countries in the final row are not EARS-Net participants and report through the WHO CAESAR network, whose country-level annual tables are not reproduced in the ECDC reports [7,28]. An independent ECDC document covering 2019 and 2023 reproduces the identical country values but a different EU/EEA aggregate (2.52 and 3.97), reflecting a different aggregation method; the two aggregates should not be presented as the same quantity [27].

### 3.2. Romania

Romania is the best-documented country in the region and the one with the highest burden. Its position was already established before the review period: national data cited in the Romanian-language literature record carbapenem resistance among invasive *K. pneumoniae* at 14–21% in the EARS-Net reporting of 2012–2013 [29], rising to 35.9% by 2016 [30] and to 54.5% by 2021, at which point Romania ranked second in the EU/EEA behind Greece [31]. The 2024 estimated bloodstream-infection incidence of 20.31 per 100,000 places it first [5].

Single-centre series confirm and extend this picture. A Bucharest infectious diseases hospital recorded CRKP prevalence among *K. pneumoniae* rising from 4.9% (2010) to 41.6% (2024), with colistin use up 186% over 2019–2024 [32]. A seven-year Bucharest bloodstream-infection series found 43.2% of *K. pneumoniae* isolates to be carbapenemase producers (57.6% multidrug-resistant overall), with colistin resistance reaching 20.9%, peaking at 28% in 2023 [33]. A 2023–2025 national institute series of 340 carbapenem-non-susceptible isolates found over 90% carbapenemase producers, 56.47% extensively drug-resistant, and a further 17.06% pandrug-resistant, with colistin resistance reaching 70.29% of the collection—the highest figure reported for Romania among unselected clinical collections in this review; a higher rate (79.8%) is reported separately below within the molecularly defined NDM plus OXA-48-like co-producer subgroup specifically (Section 4.1) [34].

Colonisation data provide a complementary measure. A five-year admission-screening programme at the same institution found carbapenemase-producing *Enterobacterales* carriage rising from 1.15% in 2015 to 6.77% in 2019, with 37% of all screened patients carrying at least one multidrug-resistant organism [35]. In an intensive care unit in Mureș County, carbapenem-resistant organisms were recovered from 4.25% of pharyngeal and 6.38% of rectal screening swabs, with *Klebsiella* and *Acinetobacter* predominating [36].

Romanian-language sources add texture the English-language literature does not supply. The 2023 national point-prevalence survey (ECDC protocol) found *K. pneumoniae* in 16.9% of healthcare-associated infections and 42.9% antimicrobial-use prevalence [37]; a Cluj regional survey of 9375 patients the same year found an HAI prevalence of 2.6%, with *K. pneumoniae* accounting for 16.8% of isolates [38]. The gap between this 2.6% regional figure and the 7.1% European crude prevalence [1] more plausibly reflects an ascertainment difference than a true epidemiological one—itself a finding about Romanian HAI surveillance maturity. National HAI incidence from the communicable disease surveillance centre was 1.04% for 2020 versus 0.63% for 2019 [31], an order of magnitude below the European estimate and consistent with substantial under-ascertainment.

Antimicrobial consumption is the other side of the Romanian picture. Romanian-language sources report total national consumption of 26.29 defined daily doses (DDD) per 1000 inhabitants per day in 2018, then the fifth highest in Europe [39], and 25.7 DDD per 1000 inhabitants per day in 2021, at which point Romania ranked first in the EU/EEA against a European average of 16.4 [31]. For context, the EU/EEA population-weighted mean in 2024 was 20.3 DDD per 1000 inhabitants per day [40].

### 3.3. Bulgaria

Bulgaria presents the region’s most dramatic recent trajectory. Its estimated CRKP bloodstream-infection incidence was 2.24 per 100,000 in 2019—below the EU/EEA figure—and 10.62 by 2024, a rise of 374.1% and the steepest in the EU/EEA [5,27]. Bulgaria therefore moved during the review period from a country with a below-average burden to one approaching the levels seen in Greece.

The national reference laboratory data recovered from the Bulgarian-language congress literature quantify the hospital-level reality behind this trend. Of 146 strains characterised by the National Reference Centre for Healthcare-Associated Infections in 2022, 72.6% (106/146) were carbapenem-resistant *K. pneumoniae*, with colistin resistance among those reaching 22.6% (24/106) [16]. A national typing study covering 465 *Klebsiella* clinical isolates from 22 hospitals and three outpatient laboratories found 67.7% to be carbapenem-resistant [41]. These proportions refer to isolates referred for national characterisation and are therefore enriched for resistance; they are not national prevalence estimates, but they establish that carbapenem resistance now dominates the referred *Klebsiella* population.

Bulgarian molecular epidemiology has changed materially during the review period. The foundational multicentre study (2014–2018, eight centres) found KPC-2 (*Klebsiella pneumoniae* carbapenemase, variant 2) (51%) and NDM-1 (New Delhi metallo-β-lactamase, variant 1) (47%) co-dominant, with NDM-1 almost entirely confined to ST11 [42]. During the pandemic, a Varna study of COVID-19 ward and intensive care isolates found *bla*KPC in 62.2%, *bla*NDM in 35.1%, and *bla*VIM in 27.0%, with 18 distinct ERIC-PCR (enterobacterial repetitive intergenic consensus PCR) profiles evidencing intrahospital dissemination under pandemic conditions [43]. By 2024, two independent Sofia studies documented a complete shift in the carbapenemase spectrum towards NDM-5 combined with OXA-232 on separate transmissible plasmids within a single ST101-like clone (ST6260), against which only cefiderocol retained activity [44,45].

Clinical consequence is documented at both ends of the care pathway. In the Sofia military hospital intensive care unit during 2023, 379 multidrug-resistant *K. pneumoniae* isolates were recovered, of which 88% were carbapenem-resistant; nine colistin-resistant isolates in six patients formed a time–space cluster, colistin resistance was *mgrB*-mediated with no *mcr* genes detected, and mortality in that group was 60% [46]. In haematology patients screened at a Varna university hospital between December 2023 and June 2024, CRKP colonisation was found in 42% of 180 patients, and among the 29 patients who subsequently developed bloodstream infection, *K. pneumoniae* was responsible in 44.8%, of which 76.9% were carbapenem-resistant and NDM-positive; 21.4% of CRKP carriers went on to develop CRKP bacteraemia [47].

One negative finding from Bulgaria is important for the region. A community faecal-carriage study of 717 samples from Sofia, Pleven, and Burgas found extended-spectrum β-lactamase (ESBL) carriage of 14.6% and plasmid-mediated AmpC carriage of 2.5%, but recovered no carbapenem-resistant isolates [48]. On this evidence, Bulgarian CRKP remained a hospital-confined phenomenon during the study period—a contrast with Croatia and Serbia, discussed below.

### 3.4. Greece

Greece has the longest-established CRKP problem in Europe and, in consequence, the most mature molecular surveillance. It is also the only country in the region whose recent trajectory is not simply upward.

The pre-pandemic Greek picture was dominated by KPC. Between 2013 and 2016, however, a survey of 480 metallo-β-lactamase-positive carbapenem-non-susceptible *K. pneumoniae* from eight Greek hospitals found *bla*NDM-1 in 341 (71%), with a strongly rising trend and a predominant ST11 clone present in all eight hospitals—a clone the authors identified as identical to that responsible for a 2015 NDM-1 outbreak in Bulgaria, one of the clearest pieces of evidence for cross-border clonal traffic within the region [49].

During and after the pandemic, three independent Greek series document a shift away from KPC monopoly. At a large Athens hospital, 2021 carbapenemase-producing *Enterobacterales* isolates collected 2020–2023 showed KPC remaining predominant each year (40.7–53.5%) but with a rapid VIM (Verona integron-encoded metallo-β-lactamase)-to-NDM transition among metallo-enzymes and resistance rising significantly post-COVID (2022–2023) across nearly all agents tested [50]. In Thessaloniki intensive care units (March 2018–March 2021), *bla*KPC (alone or with *bla*VIM) peaked at 44.4% in 2019, after which *bla*NDM rose to 45.5% (2020) and 60.7% (2021), attributed to intensive empirical use of newer agents [51]. Most strikingly, a seven-year bloodstream-infection series (671 CRKP, 2019–2025) found KPC producers at 67.4% overall—rising to 2022, then falling sharply—while dual-carbapenemase producers (17.4%) and single metallo-β-lactamase producers (15.2%) rose steadily, and colistin resistance rose continuously [52].

Dual-carbapenemase production is increasingly reported in Greece, illustrated by three recent series. In a 2023 Thessaloniki intensive care cohort of 32 non-duplicate CRKP, *bla*KPC alone and *bla*NDM alone each accounted for 25%, and both genes were co-harboured in 50%; critically, KPC-only isolates remained susceptible in vitro to ceftazidime-avibactam, meropenem-vaborbactam, and imipenem-relebactam, whereas all dual KPC-NDM producers were resistant to all three [53]. An Athens surgical and transplantation intensive care unit reported an outbreak driven simultaneously by two double-carbapenemase populations—17 ST11 isolates carrying OXA-48/NDM and four ST39 isolates carrying KPC/NDM [54]. A regional, non-tertiary Greek hospital in Volos found among 37 CRKP isolates from 2022–2024 *bla*NDM-1 in 45.9%, *bla*VIM-1 in 27.0%, and *bla*KPC-2 in 18.9%, with multiple carbapenemases co-expressed in 30% and colistin resistance at 40% [55]—evidence that the phenomenon is not confined to the Athens and Thessaloniki referral centres.

Greece also supplies the region’s clearest warning about resistance to the newer agents. An outbreak in an Athens intensive care unit involving five patients over four weeks in late 2023 and early 2024 was caused by ST323 *K. pneumoniae* co-harbouring KPC-2 and VEB-25, with ceftazidime-avibactam minimum inhibitory concentrations above 256 mg/L and cefiderocol at 32 mg/L, the latter attributable to a disrupted catecholate siderophore receptor; the authors note that ceftazidime-avibactam-resistant KPC-2 plus VEB-25 *K. pneumoniae* has been increasing in Greece over five years [56]. An ECDC molecular follow-up survey of 15 Greek hospitals in 2022 identified 44 within-hospital transmission events across 12 hospitals and found ST323—absent from earlier Greek surveys—in six hospitals [57].

Greek-language sources add two elements not available internationally. The national public health organisation’s 2023 surveillance report records *Klebsiella* spp. as the single most frequent genus in healthcare-associated infections (20.5% of all organisms), with 64% of *Klebsiella* isolates carbapenem-non-susceptible (73/114) and overall *Enterobacterales* carbapenem resistance of 46.8% (80/171) [21]. The Greek national action plan documents that the national AMR/HAI/consumption surveillance system—“Procrustes”—launched in November 2010 and became mandatory for all Greek hospitals from 2014 [22], a continuity of national reporting no other country in the region matches. A Greek doctoral thesis provides the full molecular dataset underlying the Thessaloniki intensive care series (KPC 37.3%, NDM 36.0% among 150 CRKP, June 2018–July 2021) [25], and a national paediatric surveillance report (23 units, 43 months) documented 519 central-line-associated bloodstream infections, of which 14.7% of Gram-negative isolates were carbapenem-resistant [58].

Greek antimicrobial consumption remains the highest in the EU/EEA, at 29.9 DDD per 1000 inhabitants per day in 2024 against a European mean of 20.3 [40].

### 3.5. Croatia and Slovenia

Croatia and Slovenia occupy the low-to-intermediate end of the regional gradient but illustrate two different patterns, and both possess national-language surveillance series of a quality unmatched elsewhere in the region.

The Croatian national antibiotic susceptibility series, published annually in Croatian by the Croatian Academy of Medical Sciences, reported *K. pneumoniae* imipenem resistance of 7% in 2020 rising to 11% in 2023, with meropenem resistance stable at 16% across both years; the 2023 report covers 6557 nationally reported *K. pneumoniae* isolates [17,18]. This national picture—modest absolute levels, a clear upward drift in imipenem resistance across the pandemic period—is corroborated by the ECDC estimate of Croatian CRKP bloodstream-infection incidence rising from 1.20 to 4.49 per 100,000 between 2019 and 2024 [5].

The Croatian molecular signature is distinctive within the region: OXA-48 dominance, with very little KPC or NDM. In a study of 113 CRKP isolates, OXA-48 was found in 106, NDM in seven, and KPC in only one, with IncL the dominant plasmid type and ESBLs accompanying carbapenemases in 103 isolates [59]. That study’s most consequential finding was negative: there was no significant difference in resistance profile or determinant between hospital- and community-acquired isolates, which the authors interpret as evidence that Croatian OXA-48 has fully seeded the community. Supporting evidence comes from urinary isolates in non-hospitalised elderly patients in Zagreb County, where long-term-care-facility isolates resembled Zagreb hospital isolates more closely than community isolates did, implying transmission from hospitals into long-term care [60], and from the documented emergence of OXA-48 in significant numbers among outpatient urinary isolates after 2017 [61].

Croatia also provides the region’s best-documented post-pandemic escalation in carbapenemase complexity. Eight *K. pneumoniae* and two *Enterobacter cloacae* complex isolates carrying multiple carbapenemases were detected across three Croatian hospital centres and the outpatient setting after the pandemic—OXA-48 plus NDM in five, OXA-48 plus VIM in three, OXA-48 plus KPC in two—of which only half remained susceptible to ceftazidime-avibactam and all were resistant to imipenem-relebactam [62]. Separately, the first Croatian study of cefiderocol resistance identified 31 cefiderocol-resistant CRKP isolates from hospital and outpatient settings, carrying *bla*OXA-48 in 61%, *bla*KPC in 29%, and *bla*NDM in 12.9%, with colistin retaining activity against 71% and ceftazidime-avibactam against 87% [63].

Slovenia reports the lowest carbapenem-resistance figures in the region. National EARS-Net reporting, published in Slovenian, recorded carbapenem resistance among invasive *K. pneumoniae* of 0.9% (3 of 351 isolates) in 2021 and 1.9% (7 of 372) in 2022, with third-generation cephalosporin resistance of 22% and 20.7% respectively [19,20]. Of the four invasive isolates carrying a carbapenemase in 2021, all were OXA-48 type; by 2022, among 12 isolates with a confirmed carbapenemase, the mix had broadened to NDM in three, OXA-48 group in five, VIM in one, and OXA-48 plus NDM in three [19,20]. The Slovenian genomic baseline, established from the country’s first carbapenemase-producing *Enterobacterales* outbreak, found among 32 CRKP isolates sequenced from 2014 to 2017 ten sequence types, of which ST437 was commonest at 40.6%, with *bla*OXA-48 in 53.1%, *bla*NDM-1 in 15.6%, and both in 21.9% [64].

Despite its low case burden, Slovenia contributes a methodologically important dataset, discussed further in Section 5.4: in a seven-year interrupted time-series analysis of 441,149 patient-days at a Slovenian secondary-care hospital, the mean incidence density of total multidrug-resistant organism burden rose from 4.93 to 5.81 per 1000 patient-days during COVID-19 (*p* = 0.007) while multidrug-resistant infections fell from 1.61 to 1.29 per 1000 patient-days (*p* = 0.019)—the two measures moving in opposite directions in the same institution over the same period, driven by ESBL-producing *E. coli*, ESBL-producing *K. pneumoniae*, and carbapenem-resistant *Enterobacterales* [14].

### 3.6. Serbia and the Western Balkans

Serbia possesses the richest evidence base of the non-EU countries in the region, and it points to a pattern qualitatively different from Romania’s.

The foundational Serbian genomic study examined 2298 consecutive non-replicate *K. pneumoniae* isolates from seven hospitals in five cities, of which 426 were carbapenem-resistant and 45 colistin-resistant (10.6% of the carbapenem-resistant subset). Clonal group 101 dominated, with 38 of 45 colistin-resistant isolates belonging to ST101; no *mcr* genes were found, all genomes carried mutations associated with colistin resistance, and ST101 carried *bla*OXA-48 on a novel IncFIA-IncR hybrid plasmid also bearing *bla*CTX-M-15 [65]. The pan-European EURECA genomic survey independently identified *bla*OXA-48 with ST101 as the signature combination in Serbia and Romania, distinguishing both from the KPC/ST258-512 pattern of Greece, Italy, and Spain [66].

Contemporary Serbian data confirm OXA-48 predominance: among 312 CRKP clinical isolates collected at a Belgrade tertiary hospital between January 2023 and October 2024, OXA-48-like was the most prevalent carbapenemase [67]. A Niš tertiary centre, sampling slightly earlier, found *bla*NDM in 39.3% and *bla*OXA-48 in 37.5% of 56 genotyped carbapenemase producers, with *K. pneumoniae* accounting for 71.4% of producers and cefiderocol active against 100% [68].

As in Croatia, CRKP has also been documented outside the hospital setting in Serbia. A cross-sectional study of community isolates in Belgrade between 2016 and 2020 detected carbapenemase genes in 114 of 4800 isolates (2.4%), with 70.5% clustering into ten ERIC-PCR groups [69]. Neonatal data point the same way: among 350 preterm neonates and infants discharged from a Serbian tertiary centre between April 2018 and May 2019, carbapenem-resistant *Enterobacterales* colonisation was present in 88 (25.1%), with KPC in 45 subjects and OXA-48 in 42 [70].

Data for the remaining Western Balkan jurisdictions are limited; where available, they are summarised below. Bosnia and Herzegovina has a clear molecular signature: among 87 CRKP strains collected at the Clinical Center University of Sarajevo in 2022, 85 (97.7%) were positive for a carbapenem resistance gene, with OXA-48 in 83 (95.4%), KPC in one, and NDM in one, and with ST101 the most prevalent sequence type [71]—a picture consistent with two earlier Sarajevo outbreaks in 2017 and 2018 in which all confirmed strains were *bla*OXA-48-positive [72]. Montenegro has national aggregate percentages only, with no carbapenemase or clonal characterisation since the EuSCAPE survey [73,74]. North Macedonia’s only CRKP figures are second-hand fragments reproduced in the Western Balkans gap-mapping review [7]. For Albania, no primary study of CRKP prevalence, genotype, or outcome published in the review period was identified in any language; the only Albania-specific data available are the EuSCAPE findings reproduced in [7], which record no carbapenemase producers detected among 14 isolates tested in 2013–2014, a first KPC-producing *K. pneumoniae* in 2014, and a first NDM-producing isolate in 2018, together with the observation that no national surveillance system had been established. Kosovo has no CRKP prevalence, genotyping, or clonal data whatever; the recent Kosovar literature consists of antimicrobial stewardship implementation protocols which state explicitly that stewardship programmes have not yet been implemented [75,76].

**Table 2:** Country-level evidence base and surveillance status for carbapenem-resistant *Klebsiella pneumoniae*, Romania and South-Eastern Europe, 2020–2025.

Country	Surveillance Network	National-Language Series Identified	Molecular Characterisation Available	Pre/During/Post-Pandemic Comparison Identified	Principal Evidence Gap
Romania	EARS-Net	Yes—congress abstract volumes, national and regional point-prevalence surveys, national reports [23,24,37,38]	Extensive, including WGS [77,78]	Yes, multiple [79,80,81,82,83]	No attributable-mortality estimate
Greece	EARS-Net	Yes—national agency report, national action plan, doctoral theses [21,22,25,58]	Extensive, including national ECDC follow-up survey [57]	Yes, multiple [50,51,52,84,85]	Community/long-term-care reservoir
Bulgaria	EARS-Net	Yes—national reference centre congress reports, hospital pharmacy series [16,86]	Moderate, national reference laboratory [41,44,45]	Yes [87]	Limited outcome data
Croatia	EARS-Net	Yes—annual national susceptibility series [17,18]	Extensive, single dominant research group [59,62,63]	Yes [88,89,90]	ICU-specific CRKP series after 2018
Slovenia	EARS-Net	Yes—annual national EARS-Net reports [19,20]	Yes, national reference laboratory [64]	Yes, interrupted time series [14]	No ICU-specific CRKP study after 2017
Serbia	CAESAR	Limited; national journal literature has largely ceased since 2017	Yes, including WGS [65]	Yes [91,92]	No national annual incidence series
Bosnia and Herzegovina	CAESAR	None identified 2020–2025	Yes, single centre [71,72]	Yes, ten-year series [93]	No national aggregation
Montenegro	CAESAR	None identified	None since EuSCAPE	Yes, national aggregate [73]	No carbapenemase or clonal data
North Macedonia	CAESAR	None identified	None identified	Consumption only [94]	No CRKP prevalence or genotype data
Albania	CAESAR	None identified	None identified	None identified	No primary CRKP study in any language
Kosovo	CAESAR	None identified	None identified	None identified	No CRKP prevalence, genotype, or outcome data

CAESAR, WHO Central Asian and European Surveillance of Antimicrobial Resistance; EARS-Net, European Antimicrobial Resistance Surveillance Network; WGS, whole-genome sequencing; intensive care unit (ICU). “None identified” indicates that no source meeting the criterion was retrieved by the searches described in Section 2 and should be read as absence of retrievable evidence rather than as demonstrated absence.

## 4. Molecular Epidemiology: A Region of Three Signatures

Having shown in Section 3 that resistance burden differs sharply across the region, this section turns to the carbapenemases and clonal lineages that underlie that difference, before Section 4.3 and Section 4.4 examine colistin resistance and laboratory detection specifically.

### 4.1. The Dual-Carbapenemase Belt

The most consequential molecular finding in this review is the consolidation, in the northern half of the region, of a phenotype defined by simultaneous production of a metallo-β-lactamase and an OXA-48-like enzyme. In Romania this pattern is now not an exception but the modal genotype, and the consistency across independent centres is striking: reported proportions of NDM plus OXA-48-like co-production among characterised Romanian carbapenemase producers range from 44.4% to 61.2% across six independent peer-reviewed hospital-based series (Table 3). Two further Romanian-language congress-derived data points fall outside this core range—a 24-year single-centre series reporting 58.9%, and a subgroup analysis restricted to pandrug-resistant isolates reporting 89.15%—bringing the total number of series shown in Table 3 to eight. Six independent datasets converging on this range are unusually strong evidence for a narrative review, and it establishes dual carbapenemase production as the defining Romanian phenotype rather than an emerging curiosity.

The therapeutic implication is direct and severe. Ceftazidime-avibactam is inactive against metallo-β-lactamases; in the Romanian series where susceptibility was reported alongside genotype, ceftazidime-avibactam resistance among the co-producing group reached 83.7% [98] and 100% [97]. The same isolates showed colistin resistance of 79.8% [97], and in one series NDM plus OXA-48-like isolates were colistin-susceptible in only 16.5% and cefiderocol-susceptible in 58.5% [32].

This is not a uniquely Romanian phenomenon, but its regional distribution is uneven and instructive. Greece has moved towards dual carbapenemase production during the review period, though its dual producers are typically KPC-plus-NDM or KPC-plus-VIM rather than NDM-plus-OXA-48 [52,53,54]. Croatia’s post-pandemic multiple-carbapenemase isolates carry OXA-48 in combination with NDM, VIM, or KPC [62]. Slovenia’s 2022 national reporting recorded three invasive isolates with OXA-48 plus NDM [20]. Bulgaria’s shift has taken a different form again, towards NDM-5 combined with OXA-232 on separate transmissible plasmids within a single ST101-like clone [44,45]. Carbapenemase distribution across the region is summarised in Table 4. Table 4 also reports the dominant sequence types (STs) described for each country, which is the molecular-epidemiology distribution requested by reviewers; a dedicated geographic map of ST distribution was not constructed because sequence-typing capacity and reporting are themselves unevenly distributed across the region (Section 2.4), and a map would imply a completeness the underlying data do not have. Section 4.2 discusses the three cross-border clonal lineages visible within Table 4 in narrative detail.

### 4.2. Clonal Lineages

Three clonal signatures can be distinguished across the region, and they correspond only loosely to national borders.

The first is the ST101 belt. ST101 carrying *bla*OXA-48 is the signature combination in Serbia and Romania according to the EURECA genomic survey [66], is the most prevalent sequence type among Bosnian CRKP [71], appears among carbapenem-resistant Croatian isolates [102], and, in Croatia, occurs as ST101/KPC-2 with colistin resistance in the Split region [103]. Bulgaria’s newly dominant ST6260 is an ST101-like type [44]. Romanian colistin-resistant isolates from two hospitals were predominantly ST101 (six of fourteen) followed by ST147 (five) [78].

The second is the Greek high-risk clone complex. ST258 and its derivative ST512, both predominantly KPC-carrying, remain present in Greece, and ST11, ST15, ST30, ST35, ST39, ST307, ST323, and ST512 were all recovered in a single Greek tertiary hospital between 2018 and 2022 [100]. ST323, previously unrecorded in Greek surveys, was found in six of fifteen hospitals in the 2022 ECDC follow-up survey [57] and has since been implicated in ceftazidime-avibactam- and cefiderocol-resistant outbreaks [56].

The third is displacement. The Balkan review of Chatzidimitriou and colleagues concluded that since 2018 ST11 appears to have replaced ST258 across the Balkan peninsula, while ST15 persists [6]. Bulgarian NDM-1 was almost entirely ST11 in the 2014–2018 multicentre study [42], and ST11 accounted for 57.8% of Bulgarian carbapenemase producers in a later four-hospital survey [101]. In Greece, ST11 carried the NDM-1 clonal outbreak across all eight surveyed hospitals [49].

Romania appears to be diverging from this pattern. A recent whole-genome-sequencing study of 101 carbapenem-resistant *K. pneumoniae* isolates collected between July 2023 and May 2025 at a Bucharest tertiary hospital identified ST383 as the dominant sequence type (31 isolates, 30.7%), with 29 of those 31 co-harbouring *bla*NDM-5, *bla*OXA-48-like, and *bla*CTX-M-15 on mosaic resistance–virulence plasmids showing evidence of chromosomal integration; these were the first documented Romanian NDM-5 plus OXA-48 co-harbouring strains [77]. No retrieved study reports ST258 as currently dominant anywhere in Romania.

### 4.3. Colistin Resistance

Colistin resistance is addressed here, rather than treated as a separate topic, because it determines how much treatable margin CRKP leaves once a carbapenemase has already removed the carbapenems, and in metallo-β-lactamase producers also ceftazidime-avibactam, from the therapeutic repertoire (Section 6.2): colistin and polymyxin B are among the few agents that remain, so the mechanism and trajectory of colistin resistance in the region set the outer boundary of what is still treatable in CRKP infection.

The mechanism of colistin resistance is remarkably consistent across the region, and the consistency has a practical consequence.

Chromosomal alteration, not plasmid-mediated *mcr* (mobile colistin resistance), is the dominant route. In Serbia, all 45 colistin-resistant genomes carried mutations associated with colistin resistance, all had a mutated MgrB, and no *mcr* genes were found [65]. In Bulgaria’s Sofia intensive care unit cluster, colistin resistance was *mgrB*-mediated and no *mcr* genes were detected [46]. In Romania, whole-genome sequencing of colistin-resistant and colistin-heteroresistant isolates from two hospitals found no *mcr* genes, with *mgrB* disrupted by insertion sequences in seven isolates and deleterious missense mutations in *pmrB*, *phoQ*, and *crrB*-type loci [78]. The Balkan regional review reached the same conclusion for the peninsula as a whole [6].

The exception is instructive rather than contradictory. The first human *mcr-1*-mediated colistin resistance reported in Romania was found in five of 34 colistin-resistant isolates in a study of carbapenem-resistant *Enterobacterales* urinary tract infections conducted in Iași between 2019 and 2022 [79]. That *mcr* is present in only a minority of isolates underlines the point: colistin resistance in this region is overwhelmingly a consequence of selection pressure acting on chromosomal loci within individual patients, rather than of horizontal acquisition. Since chromosomal resistance is generated by exposure, colistin resistance in the region is a direct function of colistin use—and colistin use has risen sharply, by 186% in one Romanian centre between 2019 and 2024 [32], and significantly during the pandemic in a north-eastern Romanian centre, peaking in the Omicron phase [104].

Reported colistin resistance figures across the region are correspondingly high and rising: 20.9% (peak 28% in 2023) in a Romanian bloodstream series [33], above 70% in a Romanian carbapenem-non-susceptible collection [34], 79.8% among Romanian NDM plus OXA-48 co-producers [97], 22.6% among Bulgarian nationally referred CRKP [16], 40% in a Greek regional hospital [55], and rising continuously in a seven-year Greek bloodstream series [52].

### 4.4. Implications for Laboratory Detection

The sources cited in this review establish carbapenem resistance and carbapenemase production by two distinct routes: phenotypically, through disk diffusion or automated minimum-inhibitory-concentration testing interpreted against a breakpoint standard, sometimes supplemented by a confirmatory phenotypic carbapenemase assay; and genotypically, through PCR-based gene detection, lateral-flow immunochromatographic assays, or whole-genome sequencing (WGS is the method underlying the sequence-type assignments in Table 4). Genotypic methods establish carbapenemase gene content directly and are not breakpoint-dependent; supranational surveillance (ECDC/EARS-Net, Table 1) defines carbapenem resistance phenotypically using EUCAST breakpoints, and EUCAST is the standard used, where stated, by most other phenotypic testing cited in this review, with CLSI reported explicitly only where it materially changed the result, as in the Bulgarian cefiderocol comparison in Section 6.2, where susceptibility was 37.5% by EUCAST against 71.8% by CLSI criteria for the same isolates [101]. A complete, per-country tabulation of phenotypic versus genotypic method and breakpoint standard for every study cited was not attempted, because most of the national-language congress abstracts and agency reports retrieved for this review do not state which standard was applied; where a source does specify its method, that method is named in the text or table alongside the finding (for example, the WGS-based sequence types in Table 4, and the immunochromatographic assay sensitivity and specificity figures discussed below), and its absence from a country’s entry indicates that the retrieved record did not report it, not that no CRKP was detected.

Two detection problems follow from the molecular picture described above, and both bear directly on the region.

The first is phenotypic under-ascertainment of OXA-48-like producers. OXA-48-like enzymes hydrolyse carbapenems weakly, and isolates may remain susceptible to imipenem and meropenem while resistant to ertapenem. Croatian data illustrate this precisely: among 46 colistin-resistant *K. pneumoniae* isolates from five hospitals and the community, most OXA-48 producers were ertapenem-resistant but imipenem- and meropenem-susceptible [105]. In Slovenia, one of the four invasive isolates carrying a carbapenemase in 2021 remained susceptible to both imipenem and meropenem [19]. In a region where OXA-48-like enzymes dominate in Croatia, Serbia, and Bosnia and are half of the Romanian dual-producer phenotype, screening algorithms that rely on imipenem or meropenem non-susceptibility as the trigger will systematically under-detect the transmissible reservoir.

The second is that carbapenemase identification, not merely carbapenemase detection, has become therapeutically decisive. The Greek intensive care data make this concrete: KPC-only isolates were susceptible in vitro to ceftazidime-avibactam, meropenem-vaborbactam, and imipenem-relebactam, while dual KPC-NDM producers from the same unit were resistant to all three [53]. Distinguishing between them changes first-line therapy. Lateral-flow immunochromatographic assays that identify the five commonest carbapenemase families directly from culture perform well in regional evaluations: a Belgrade study of 312 CRKP isolates found sensitivity of 98.7% and specificity of 100%, although the assay failed to detect VIM in five PCR-positive isolates [67], and a Romanian medico-legal evaluation of the same assay class on post-mortem samples achieved 92.3% sensitivity and 100% specificity against pure culture [106]. Non-carbapenemase mechanisms should not be forgotten: an outbreak in Rijeka was caused by ertapenem-resistant, carbapenemase-negative, porin-deficient ESBL-producing *K. pneumoniae* [107], and proteogenomic analysis including Romanian isolates has shown that increased carbapenemase plasmid copy number combined with OmpK36 loss can drive high carbapenem minimum inhibitory concentrations [108].

## 5. The Pandemic Transition: Before, During, and After

The regional and molecular landscape described in Section 3 and Section 4 sets the context for the review’s second objective, addressed here: how the pandemic altered these patterns, and whether the alteration proved transient or persistent.

### 5.1. What the Surveillance Series Show

Read as a whole, the regional surveillance data describe a sequence rather than a single event: a brief interruption of pre-existing upward trends in 2020, a steep acceleration from 2021, and persistence of the elevated level after 2022.

The ECDC incidence series in Table 1 shows this clearly for Bulgaria (2.24 in 2019, 2.19 in 2020, then 3.52, 3.91, 7.75, and 10.62), for Croatia (1.20, 1.57, 2.87, 2.52, 4.53, 4.49), and for Romania (7.12, 10.77, 13.87, 12.12, 20.02, 20.31) [5]. In each case the largest single-year increase falls in 2022–2023, that is, after the acute phase of the pandemic rather than during it. Greece is the exception already noted: peak in 2021, partial retreat thereafter [5,27]. The Croatian national series shows *K. pneumoniae* imipenem resistance moving from 7% in 2020 to 11% in 2023 [17,18]; the Slovenian national series shows invasive carbapenem resistance doubling from 0.9% in 2021 to 1.9% in 2022, with the carbapenemase mix broadening from OXA-48-only to a four-enzyme picture over the same twelve months [19,20].

Antimicrobial consumption follows a related but distinct trajectory. At EU/EEA level, total consumption fell temporarily in 2020 and 2021 and had returned to slightly above the 2019 baseline by 2023 [109]. National data show the same dip-and-rebound: Croatian outpatient prescribing fell 21.0% in 2020 and had returned by 2024 to a level virtually identical to pre-pandemic [88]; Slovenian national community consumption fell 23.5% in 2020 and 24.3% in 2021 [110]. Hospital consumption, however, moved in the opposite direction. Bulgarian-language data record hospital consumption doubling in 2020, the first pandemic year, directly counter to the EU-wide community decline [86]. In a North Macedonian intensive care unit, total antibiotic use fell between 2020 and 2021 while carbapenem consumption rose from 1.7% to 14.37% of the total [94]. This divergence—community prescribing down, hospital and specifically carbapenem prescribing up—is the mechanistic bridge between the pandemic and the resistance trajectory.

### 5.2. Three-Period Studies from the Region

A number of studies published during the review period were explicitly designed around a pre-pandemic, pandemic, and post-pandemic comparison; twenty-one such studies are summarised in Table 5, spanning the nine countries of the review’s region together with one Slovakian series included as an external regional comparator. Two findings stand out. Montenegro’s national series provides the cleanest CRKP-specific comparison in the region, with explicit denominators showing carbapenem resistance rising from 17% to 47% between 2019 and 2022 [73]. Serbia’s blood-culture series documents one of the sharpest pandemic-to-post-pandemic transitions anywhere in the region, with *K. pneumoniae* prevalence roughly stable through 2020–2021 before more than doubling in 2022 [92]. Beyond these two, the pattern across the table is consistent: resistance indicators typically peak in 2022–2023, after rather than during the acute pandemic phase, while a case-mix shift towards sicker, more heavily instrumented patients, documented explicitly in the Timișoara paediatric series [83], complicates the interpretation of any single proportion in isolation.

### 5.3. Mechanisms

The mechanistic literature is now sufficiently developed to explain the sequence described above without recourse to speculation.

The proximate driver is antibiotic exposure disproportionate to bacterial infection. Meta-analysis found bacterial co-infection at COVID-19 presentation in 3.5% of patients and secondary bacterial infection in 14.3% [10], against antibiotic prescribing in 74.6% [11]. A multicentre point-prevalence study conducted in seven tertiary university hospitals in Croatia, Italy, Serbia, and Slovenia—four of the countries considered here—found that 521 of 988 hospitalised COVID-19 patients (52.7%, hospital range 32.9–85.6%) were receiving antibiotics or antifungals on the survey day, 79.4% of prescriptions were empirical, and 69.9% of antibiotics belonged to the WHO AWaRe (Access, Watch, Reserve) Watch group [112]. Romanian data are more extreme still: in a north-eastern Romanian COVID-19 intensive care unit, 100% of 184 patients received at least one antibiotic in the unit, only 22.3% received a single agent, imipenem was given to 75.5%, and only 9.8% had microbiological confirmation of bacterial infection [113].

That exposure has demonstrable downstream consequences. In a population-wide Ontario cohort of 53,533 non-hospitalised adults aged 66 and over, peri-COVID antibiotic receipt was associated with an adjusted odds ratio of 1.24 for any subsequent resistant organism and 1.27 specifically for Gram-negative resistant organisms within six months, with an attributable fraction among the treated of 17% [114].

The second driver is the failure of infection prevention and control (IPC) under caseload pressure. An Italian intensive care unit with an established stewardship programme and active surveillance since 2016 saw carbapenem-resistant *Enterobacterales* acquisition incidence rise from 6.7% in 2019 to 50% in March–April 2020; the authors attribute this to the intensity of care, prone positioning requiring four to five personal-protective-equipment-encumbered staff in prolonged patient contact, and the arrival of 32 redeployed healthcare workers with no intensive care experience [13]. A systematic review of multidrug-resistant organism outbreaks during the pandemic found the largest gaps between identified risk factor and implemented countermeasure to be personal protective equipment shortage and patient overcrowding, the latter identified as a risk in five outbreaks and addressed in none [12]. In a Croatian dedicated COVID-19 hospital, 39% of 118 intensive care patients developed ventilator-associated pneumonia or bloodstream infection with total mortality of 70%, and the authors explicitly link the spread to intensive care overcrowding [89]; in Croatia’s national COVID-19 hospital, 55.3% of critically ill patients developed a bacterial or fungal superinfection, with multidrug-resistant pathogens the commonest cause and significantly higher mortality among those patients [90].

A mathematical model separating these two forces quantifies their opposition. Policy responses alone—masking, patient lockdown, reinforced hand hygiene—produced a 28.2% reduction in incident multidrug-resistant colonisation; caseload responses alone—abandonment of stewardship, disorganisation of IPC programmes, extended length of stay—produced a 13.8% increase in colonisation incidence and a 10.4% increase in the antibiotic resistance rate. Combined, the model predicted colonisation incidence falling by 24.2% while the resistance rate rose by 2.9% [15].

A third factor, specific to the eastern half of the region, is cross-border patient movement. The ECDC has documented importation of carbapenem-resistant *Enterobacterales* into multiple EU/EEA countries in relation to patients from Ukraine after 2022 [27]. A systematic review of pandemic-era outbreaks reported a carbapenem-resistant *A. baumannii* cluster presumed to originate from COVID-19 patients transferred from the Balkans [12], and an analysis of conflict-zone medical evacuations compared Ukrainian antibiograms directly against national surveillance from Poland, Slovakia, Hungary, and Romania [115].

### 5.4. Why Incidence and Proportion Can Move in Opposite Directions

The Slovenian interrupted time-series result described in Section 3.5—burden up, infections down, in the same hospital over the same period [14]—is not an anomaly, and understanding it is essential to reading the regional literature correctly.

The German national analysis makes the same point with a counterfactual design. Applying Poisson and negative-binomial projection to two independent national datasets, CRKP notifications were 35% below projection in 2020, 31% below in 2021, and 6% above in 2022, with the result holding after adjustment for fewer inpatients, seasonality, and restriction to infections; the authors conclude that a genuine reduction occurred in 2020–2021 and that rising international mobility partly explains the 2022 increase [116]. A Brazilian before-and-after analysis of 9780 mechanically ventilated adults across eight hospitals found isolation of carbapenem-resistant Gram-negatives rising during the COVID-19 surge (relative risk 1.8), falling after it (0.72), but remaining above pre-pandemic levels (1.3), with *K. pneumoniae* resistance doubling during the surge and staying elevated while *P. aeruginosa* resistance reverted; culturing rates rose across periods, partially controlling for ascertainment [117].

The reconciliation is straightforward. During the acute pandemic phase, elective activity collapsed, admissions fell, and enhanced hygiene and isolation reduced transmission opportunities—so the number of resistant infections per unit population fell. At the same time, the patients who remained in hospital were sicker, more heavily instrumented, more heavily antibiotic-exposed, and cared for under degraded infection-control conditions—so the proportion of isolates that were resistant rose. When admissions returned to normal, the elevated proportion was applied to a restored denominator, and absolute incidence rose sharply. This is precisely the shape seen in the Romanian, Bulgarian, and Croatian ECDC series, where the steepest increases fall in 2022–2023 rather than 2020–2021 [5].

The practical consequence for this literature is that a study reporting a pandemic-era fall in resistant infections and a study reporting a pandemic-era rise in resistance may both be correct and may even describe the same institution. Any regional comparison must therefore state which quantity is being compared.

## 6. Clinical Outcomes and Therapeutic Options

The epidemiological, molecular, and temporal findings described above translate directly into the clinical and therapeutic consequences examined in this section.

### 6.1. Outcomes

The most conspicuous gap in the regional evidence base is mortality. Despite an extensive search in seven languages, no study published between 2020 and 2025 was identified that reports a CRKP-attributable or CRKP-associated mortality estimate for any country in this region using a design permitting comparison with carbapenem-susceptible controls. What exists instead is a set of proxy measures, and they are consistent in direction.

Intensive care admission is the dominant risk multiplier, and mortality in the region’s largest reported cohorts exceeds 50% (Table 6). Two independent Romanian cohorts converge on intensive care exposure, previous ward transitions, and NDM-type carbapenemase as the principal predictors of poor outcome, with no antibiotic regimen proving superior against NDM plus OXA-48-like isolates [95,96].

Colonisation is a strong predictor of subsequent disease: rectal carriage raised the risk of a same-species bloodstream infection five-fold in a large Greek cohort, and roughly one in five Bulgarian haematology carriers progressed to CRKP bacteraemia (Table 6) [47,119].

COVID-19 co-infection compounded outcomes throughout the region, roughly doubling intensive care admission and case fatality in one Romanian cohort and increasing the odds of death more than fifteen-fold after superinfection in another; a Serbian intensive care series of clonally related multidrug-resistant *A. baumannii* recorded 100% mortality (Table 6) [79,120,121].

Resource consumption has been quantified only sparsely in the region; the single available estimate, from a Bulgarian neonatal intensive care unit, attributes 14 additional bed-days and a near doubling of hospital cost to ventilator-associated pneumonia (Table 6) [122].

### 6.2. The Therapeutic Landscape and the Access Problem

The therapeutic position in this region cannot be read off international guidance, because the guidance assumes a carbapenemase distribution that does not obtain here.

Where KPC or OXA-48 occurs alone, ceftazidime-avibactam remains the preferred agent, with meropenem-vaborbactam and imipenem-relebactam as alternatives against KPC; regional in vitro data from Greece and Serbia confirm this holds in practice (Table 7) [53,68].

Where a metallo-β-lactamase is present, and particularly where it co-occurs with OXA-48-like, the picture inverts: resistance to ceftazidime-avibactam, imipenem-relebactam, and related β-lactam/β-lactamase-inhibitor combinations among dual producers is consistently high across Romanian, Croatian, and Greek series (Table 7) [53,62,97,98].

Two options remain, and both are supported by regional data. The first is the combination of aztreonam with ceftazidime-avibactam, which exploits aztreonam’s stability to metallo-enzymes together with avibactam’s inhibition of the accompanying serine enzymes; regional synergy and clinical outcome data are consistent and favourable (Table 7). The combination has since been formalised as fixed aztreonam-avibactam, evaluated against meropenem in the multinational REVISIT trial [124].

The second is cefiderocol. Regional susceptibility figures vary widely by series and by breakpoint standard (Table 7), a spread that itself illustrates a methodological pitfall directly relevant to regional comparability: in one Bulgarian collection, cefiderocol susceptibility was 37.5% by European Committee on Antimicrobial Susceptibility Testing (EUCAST) criteria against 71.8% by Clinical and Laboratory Standards Institute (CLSI) criteria for the same isolates [101]. Croatia has already documented cefiderocol-resistant CRKP in both hospital and outpatient settings [63], and the Greek ST323 outbreak strain combined ceftazidime-avibactam resistance with a cefiderocol minimum inhibitory concentration of 32 mg/L [56].

Colistin and polymyxin B therefore remain in use as last-resort options across much of the region, and the resulting self-reinforcing cycle is visible in the data: constrained access to newer β-lactam/β-lactamase inhibitor combinations increases reliance on colistin, and rising colistin consumption selects for the chromosomal resistance mechanisms described in Section 4.3, eroding the last-resort agent itself. The 186% rise in colistin use at one Romanian centre between 2019 and 2024, alongside falling susceptibility in 2023–2024 [32], and the peak of colistin use in the Omicron phase at another [104], document this cycle in progress.

Access is not a purely microbiological question. Reimbursement pathways and procurement processes for ceftazidime-avibactam, aztreonam-avibactam, and cefiderocol differ substantially across the health systems considered here, and the systematic review of Eastern European carbapenem-resistant *Enterobacterales* explicitly flags the scarcity of real-world regional treatment data as a barrier to establishing what is actually being used [8]. Serbia’s national antibiotic resistance control programme covered 2019–2021 and no successor plan for the subsequent period was retrievable [125]; Kosovo has no implemented antimicrobial stewardship programme in primary care [75,76]; Albania had no national surveillance system established at the time of the most recent regional assessment [7]. Where stewardship has been implemented, it works: a carbapenem-focused programme introduced during the pandemic at a Cretan university hospital reduced the proportion of patients receiving carbapenems from 4.1% to 2.3% and carbapenem use by 4.9 DDD per 100 patient-days, with 30-day infection-related readmission falling from 24.6% to 16.8% and no increase in mortality [126].

## 7. One Health Dimensions

Environmental data from the region are sparse but consistent, and they close the loop between hospital antibiotic use and community exposure.

Croatian work provides the most complete picture. In treated wastewater from the Zagreb plant, 148 confirmed *Enterobacterales* were recovered from 200 suspected carbapenem-resistant isolates, *Klebsiella* spp. being the predominant genus at 47 isolates; all 148 were carbapenemase producers with a multidrug-resistant phenotype, spanning 18 sequence types of which 14 were associated with human clones [127]. In hospital wastewater from two large Zagreb hospitals, 69 ESBL-producing and 90 carbapenemase-producing *Enterobacterales* were recovered, all multidrug-resistant [128]. Downstream of that, KPC- and OXA-48-producing *Enterobacteriaceae* have been recovered from Central Adriatic coastal waters receiving submarine effluent from two wastewater treatment plants, with whole-genome sequencing identifying 137 resistance genes across 19 drug classes and four isolates co-harbouring two carbapenemase genes [129].

Romanian environmental data are more fragmentary but point the same way. Antibiotic residues and resistance were characterised in influent and effluent from three Romanian wastewater treatment plants across four seasons in 2021–2022, with several antibiotics persisting in effluent and higher bacterial resistance recovered in warmer seasons [130]. The national RADAR project, described in the Romanian-language literature, samples wastewater treatment facilities in seven Romanian cities—Bucharest, Timișoara, Iași, Galați, Constanța, Cluj, and Râmnicu Vâlcea—targeting *K. pneumoniae* among other priority organisms, with the explicit aim of correlating hospital antibiotic consumption with environmental resistance levels [131]. A three-country study including Romania was designed to sample air, water, and sewage inside and outside treatment plants alongside stool from plant workers, nearby residents, and a distant comparison group, targeting ESBL-producing *E. coli* and carbapenemase-producing *K. pneumoniae* [132]. Bulgarian metagenomic work downstream of a wastewater treatment plant serving 125,000 people detected *bla*OXA-58 and *bla*IMP-33-like genes in surface water, although the calculated human health risk score was low [133].

Two implications follow. First, the wastewater compartment can be read as a surveillance signal rather than only as a hazard, particularly in countries where clinical surveillance is incomplete—a point of some importance for the Western Balkan jurisdictions absent from EARS-Net. Second, the Croatian finding that hospital- and community-acquired *K. pneumoniae* isolates no longer differ in resistance profile or determinant [59], read alongside the Serbian detection of carbapenemase genes in 2.4% of community isolates [69], suggests that in at least two countries of the region the hospital–community boundary has already been crossed. The Bulgarian community carriage study, which found no carbapenem-resistant isolates [48], indicates that this crossing is not yet universal—which makes it a preventable event rather than an inevitability.

The hospital environment itself remains a reservoir. Romanian work has characterised biofilm formation by carbapenemase-producing *K. pneumoniae* on medical surfaces and devices and evaluated photocatalytic nanocomposite coatings as a control strategy [134], and has compared adenosine triphosphate bioluminescence against microbiological swab culture for surface sanitation assessment in a paediatric hospital [135]. Bosnian work has shown that ESBL-producing *K. pneumoniae* strains from Mostar survived on dry cotton for up to 49 days [136]—a persistence figure with direct implications for textile handling and environmental cleaning protocols.

## 8. Discussion

This section synthesises the findings presented in Section 3, Section 4, Section 5, Section 6 and Section 7 against the two objectives set out in Section 1, before turning to the limitations of a non-systematic, single-reviewer narrative synthesis.

### 8.1. Interpretation of the Evidence

Four themes emerge consistently from the evidence assembled here.

The first is that South-Eastern Europe is not epidemiologically homogeneous, and treating it as a single high-resistance bloc obscures more than it reveals. The thirty-three-fold spread in estimated CRKP bloodstream-infection incidence between Slovenia and Romania in 2024 [5] is larger than the spread between many pairs of continents. The molecular pictures differ correspondingly: Croatia, Serbia, and Bosnia are OXA-48 territories with limited metallo-β-lactamase penetration [59,65,71]; Greece is a KPC territory in transition towards metallo-enzymes and dual production [50,51,52]; Romania and, more recently, Bulgaria are dual-carbapenemase territories [34,44,77,95,96,97,98,99]. These are not gradations of a single problem but different problems requiring different empirical therapy.

The second is that the dual-carbapenemase phenotype has become the defining feature of the Romanian epidemiological landscape and is the single most consequential finding for clinical practice in the region. Six independent Romanian centres, using different methods across different periods, report metallo-β-lactamase plus OXA-48-like co-production in 44–61% of characterised carbapenemase producers [34,77,95,96,97,98,99]. Because ceftazidime-avibactam is inactive against metallo-enzymes, this phenotype removes the agent that international guidance identifies as first-line, and it does so not in a minority of difficult cases but in the majority of characterised isolates. Empirical therapy for suspected CRKP infection in Romania therefore cannot be transposed from guidance written for settings where single-carbapenemase producers predominate, and must instead be anchored on aztreonam-avibactam or cefiderocol pending rapid carbapenemase identification.

The third is that the pandemic’s effect on regional CRKP was neither a simple increase nor a simple interruption, but a phase shift. The pattern visible across the surveillance series—transient dip, steep rise from 2021, persistence after 2022 [5,17,18,19,20]—matches what the mechanistic literature predicts when a temporary reduction in transmission opportunity is superimposed on a durable increase in selection pressure and a durable degradation of infection prevention capacity [15]. The most important corollary is that the pandemic-era changes did not reverse. In every country in the region except Greece, the 2024 incidence is at or near the highest value recorded in the six-year series [5]. Whatever occurred between 2020 and 2022 has been retained.

The fourth is that the national-language literature is not merely supplementary but load-bearing for the parts of the region least visible internationally. The Bulgarian national reference centre’s 2022 figure of 72.6% carbapenem resistance among referred *K. pneumoniae* with 22.6% colistin resistance [16] has no English-language equivalent. The Croatian and Slovenian national susceptibility series [17,18,19,20] supply annual national denominators that no PubMed-indexed source provides. The Romanian congress abstract volumes contain the only Romanian-language pre- versus post-pandemic CRKP comparison identified—carbapenem-resistant *Enterobacterales* rising from 27.6% of *K. pneumoniae* in 2018 to 34.8% in 2022 at a Bucharest hospital, with colistin resistance of 52% among carbapenemase producers [23]—together with the national and regional point-prevalence survey results [37,38]. The Greek national surveillance report gives a *Klebsiella* share of healthcare-associated infections and a carbapenem non-susceptibility proportion that appear in no English-language source [21].

### 8.2. What Is Contested and What Is Missing

Three areas warrant caution.

The most serious is mortality. No CRKP-attributable mortality estimate for any country in this region was identified for the review period. The proxies are consistent and alarming—51.79% mortality among Romanian patients with multidrug-resistant HAIs [118], 60% in a Bulgarian colistin-resistant intensive care cluster [46], a 40.8-fold in-hospital death risk associated with intensive care stay in a Romanian CRKP cohort [95]—but none of these is an attributable-mortality estimate, and none permits comparison against carbapenem-susceptible controls in the same setting. This is a substantial gap in a region that carries Europe’s highest burden.

The second is comparability of resistance figures. The Romanian trajectory can be assembled from verified sources as 14–21% (EARS-Net 2012–2013) [29], 35.9% (2016) [30], 54.5% (2021) [31] at national level, alongside single-centre figures of 27.6% (2018) and 34.8% (2022) [23], 41.6% (2024) [32]. The whole-genome-sequencing series [77] characterised only isolates already confirmed as carbapenem-resistant and therefore does not itself report a prevalence-trajectory percentage comparable to these three; no such figure from that series is carried forward here. These cannot be presented as a single continuous series: the national figures use invasive-isolate denominators, the single-centre figures use all-isolate or referred-isolate denominators, and the highest values come from collections enriched by referral for molecular characterisation. Presented separately, they nonetheless tell a consistent story of sustained increase.

The third is the surveillance void in the Western Balkans. Six jurisdictions covering roughly 17 million people generate no comparable annual incidence data [7,28]. Serbia partially compensates through a strong research literature [65,67,69,91,92]; Bosnia has a clear molecular signature from Sarajevo [71,72] and a ten-year invasive-isolate series from Banja Luka [93]; but Montenegro has national aggregates without molecular characterisation [73,74], North Macedonia has almost nothing beyond consumption data [94], and Albania and Kosovo have essentially no primary CRKP data at all [7,75,76]. Absence of data from these countries should not be read as absence of CRKP.

### 8.3. Limitations

This review has limitations falling into three groups.

The first concerns methodology. It is a narrative rather than a systematic review: study selection was not performed in duplicate, no formal risk-of-bias instrument was applied, and no quantitative pooling was undertaken. Selection of studies was guided by relevance, methodological quality, and the need for country-level coverage, and this introduces the possibility of selection bias in favour of countries and centres that publish more.

The second concerns the multilingual search. Although the national-language search was systematic in intent, it was constrained in execution. Several key national repositories—including the Romanian national public health institute, the Bulgarian national reference laboratory journal, the WHO European Health Information Gateway, and the Croatian national repository—restrict automated retrieval, so their contents were confirmed to exist but could not in every case be read in full. Where figures from such sources are quoted here, they are quoted from secondary national-language reports that cite them, and this is indicated in the text. Furthermore, several national-language sources are congress abstracts rather than peer-reviewed full papers; their figures are reported as published but have not undergone full peer review and complete bibliographic fields could not always be established. No Bosnian-, Macedonian- or Albanian-language scientific source on CRKP published between 2020 and 2025 was identified, which may reflect either absence of such literature or limitations of the search rather than absence of the problem.

The third concerns the evidence itself. Molecular characterisation capacity is unevenly distributed, so the carbapenemase and clonal distributions in Table 4 partly reflect where sequencing is performed rather than where organisms circulate. Several of the pandemic-comparison studies are single-centre and were not designed with a formal interrupted time-series or counterfactual approach, so pandemic effects cannot be cleanly separated from secular trends. Case-mix change during the pandemic—documented explicitly in the Romanian paediatric data, where admissions halved and the specimen mix shifted materially [83]—is a plausible confounder of every pandemic-era resistance proportion reported here, and few of the primary studies adjust for it.

### 8.4. Implications for Practice, Policy and Research

For clinical practice, the central implication is that empirical therapy for suspected CRKP infection must be guided by local molecular epidemiology and supported by rapid carbapenemase identification rather than carbapenemase detection alone. In Romanian and, increasingly, Bulgarian settings, the working assumption for a critically ill patient with suspected CRKP infection should be that a metallo-β-lactamase is present until shown otherwise, with aztreonam-avibactam or cefiderocol as the empirical anchor. In Croatian, Serbian, and Bosnian settings, where OXA-48 predominates, ceftazidime-avibactam remains appropriate—but the ertapenem-susceptible, imipenem- and meropenem-resistant phenotype pitfall means screening algorithms must not rely on imipenem or meropenem non-susceptibility alone [105].

For infection prevention, the pandemic experience establishes that the two forces acting on transmission are separable and act in opposite directions [15]. The policy components that reduced transmission—hand hygiene reinforcement, isolation discipline, reduced patient movement—are retainable in normal times; the caseload components that increased it—stewardship suspension, staff redeployment without training, overcrowding—are avoidable with adequate surge planning. That patient overcrowding was identified as an outbreak risk factor in five of seventeen reviewed outbreaks and addressed in none [12] is a specific and actionable failure.

For policy, the surveillance void in the Western Balkans is the priority. Extending molecular characterisation capacity and reporting continuity to Montenegro, North Macedonia, Albania, and Kosovo would cost little relative to the burden it would illuminate, and the Croatian and Slovenian national report series demonstrate that a small country can produce high-quality annual national data with modest resources [17,18,19,20]. Sustaining national action plans matters equally: Serbia’s programme lapsed after 2021 without a retrievable successor [125].

For research, four questions are open. First, an attributable-mortality study of CRKP in a high-incidence regional setting, with carbapenem-susceptible controls, is overdue. Second, the durability of the dual-carbapenemase phenotype—whether NDM plus OXA-48-like co-production is a stable endpoint or an intermediate state en route to something worse—requires longitudinal genomic surveillance rather than repeated cross-sectional description. Third, real-world outcome data for aztreonam-avibactam and cefiderocol in this region, where dual producers dominate, are almost entirely absent [8]. Fourth, the cross-border clonal traffic suggested by the Greek–Bulgarian ST11 identity [49], the Serbian–Romanian ST101/OXA-48 signature [66], and the documented importation from Ukraine [27,115] merits a coordinated regional genomic study of the kind that EuSCAPE and EURECA established as feasible.

## 9. Conclusions

Romania and South-Eastern Europe carry Europe’s highest burden of carbapenem-resistant *Klebsiella pneumoniae* in healthcare-associated infection, but the region is not uniform: estimated bloodstream-infection incidence in 2024 spanned a thirty-three-fold range within it, and three distinct molecular signatures—OXA-48 predominance in the Western Balkans and Croatia, KPC with a metallo-β-lactamase transition in Greece, and metallo-β-lactamase plus OXA-48-like co-production in Romania and increasingly Bulgaria—divide it into zones requiring different empirical therapy. The dual-carbapenemase phenotype now accounts for the majority of characterised Romanian isolates and removes ceftazidime-avibactam from first-line use, leaving aztreonam-avibactam and cefiderocol as the principal remaining options at a point where cefiderocol resistance is already documented in Croatia and Greece. The COVID-19 pandemic did not create these problems but accelerated them and made the acceleration permanent: across the region, the steepest increases fall in 2022–2023, after the acute phase, and 2024 incidence stands at or near the six-year maximum in every country except Greece. The national-language literature—congress abstract volumes, national reference laboratory reports, and agency susceptibility series in Romanian, Bulgarian, Greek, Croatian, and Slovenian—supplies data unavailable in the international databases and should be regarded as a necessary component of any regional synthesis, not an optional supplement. Two gaps demand attention: no country in the region has published a CRKP-attributable mortality estimate for the review period, and six Western Balkan jurisdictions covering some 17 million people generate no comparable surveillance data at all.

## Figures and Tables

**Figure 1:**
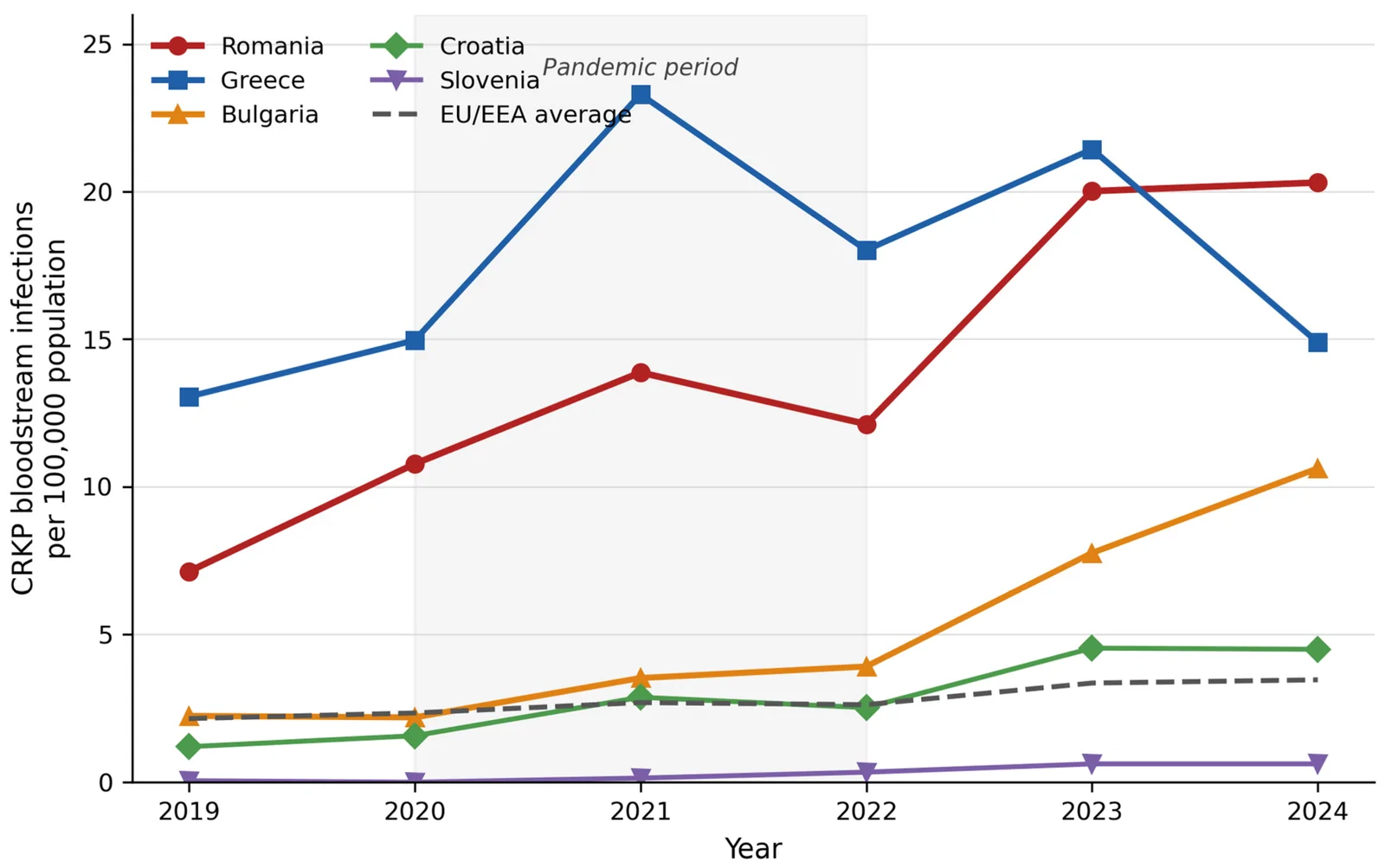
Estimated incidence of carbapenem-resistant *Klebsiella pneumoniae* bloodstream infections per 100,000 population, 2019–2024, for the five EARS-Net-reporting countries of South-Eastern Europe and the EU/EEA average. Shaded band marks the pandemic period (2020–2022). Data reproduced from Table 1 [5].

**Table 3:** Reported proportion of NDM plus OXA-48-like carbapenemase co-production among characterised carbapenem-resistant *Klebsiella pneumoniae* producers, Romania, 2020–2025.

Setting/Study	Period or Cohort	NDM + OXA-48-like Co-Production	Ref.
Bucharest emergency hospital	—	44.4%	[95]
Infectious diseases hospital cohort	22 months	49.4%	[96]
National Institute of Infectious Diseases series	2 years	53.6%	[34]
Bucharest tertiary centre, 2024 admissions	2024	55.2%	[97]
Whole-genome-sequencing study	—	55.4%	[77]
One-year prospective survey	1 year	61.2%	[98]
Single-centre series (Romanian-language congress data)	24 years	58.9%	[24]
Pandrug-resistant isolates specifically	—	89.15%	[99]

Percentages are proportions among characterised carbapenemase-producing isolates within each cited series, not national prevalence estimates. Series are independent (different centres, periods or patient groups) unless otherwise noted.

**Table 4:** Reported carbapenemase distribution and predominant clonal lineages among carbapenem-resistant *Klebsiella pneumoniae*, by country, as described in sources published 2020–2025.

Country	Predominant Carbapenemase(s)	Co-Production Pattern	Clonal Lineages Reported	Key Sources
Romania	NDM plus OXA-48-like	Dominant: 44.4–61.2% of characterised producers across six centres	ST383 (newly dominant, NDM-5), ST101, ST147, ST258, ST307	[34,77,78,95,96,97,98,99]
Greece	KPC-2, with transition towards NDM and dual production	Dual KPC/NDM up to 50% in ICU cohorts	ST258, ST512, ST11, ST15, ST39, ST307, ST323	[50,51,52,53,54,55,56,57,100]
Bulgaria	Historically KPC-2 and NDM-1; recent shift to NDM-5 plus OXA-232	NDM-5 with OXA-232 in 72% of colistin-resistant isolates in one centre	ST11, ST340, ST258, ST101, ST6260 (ST101-like)	[42,43,44,45,46,101]
Croatia	OXA-48	Multiple carbapenemases documented post-pandemic (OXA-48 with NDM, VIM or KPC)	ST101, ST14, ST15	[59,62,63,102,103]
Slovenia	OXA-48 group, with NDM increasing	OXA-48 plus NDM in 3 of 12 characterised invasive isolates in 2022	ST437, ST147, ST15	[19,20,64]
Serbia	OXA-48-like	Not prominent in retrieved data	ST101 (dominant), ST437, ST340, ST336, ST258	[65,66,67,68,69]
Bosnia and Herzegovina	OXA-48 (95.4% of characterised isolates)	Not reported	ST101 (dominant), ST307	[71,72]
Montenegro	Not characterised since EuSCAPE	Not reported	Not reported	[7,73,74]
North Macedonia	Not characterised	Not reported	Not reported	[7]
Albania	Not characterised; first KPC 2014, first NDM 2018	Not reported	Not reported	[7]
Kosovo	Not characterised	Not reported	Not reported	[7]

Entries summarise associations reported in the cited sources and do not represent a pooled quantitative analysis. Reporting intensity and molecular characterisation capacity differ markedly between countries, so absence of a genotype from this table indicates that it was not assessed or not described in the sources retrieved, rather than demonstrated absence from that country.

**Table 5:** Studies from Romania and South-Eastern Europe with an explicit pre-pandemic, pandemic, and/or post-pandemic comparison, 2020–2025.

Country, Setting	Periods Compared	Principal Finding	Source
Romania, Bucharest tertiary hospital	2019 vs. 2020–2021	Resistance rose for third-generation cephalosporins, carbapenems, aminoglycosides, fluoroquinolones, and colistin; fell for trimethoprim-sulfamethoxazole (45.7% to 28.3%)	[80]
Romania, Iași, CRE urinary tract infection	2019–2022, pre-pandemic vs. pandemic	82.7% carbapenemase producers, mainly OXA-48; COVID-19 co-infection associated with ICU admission 66.7% vs. 18.2% and case fatality 33.3% vs. 12.1%	[79]
Romania, Bucharest, single centre	2010–2024	CRKP prevalence 4.9% to 41.6%; colistin use +186% between 2019 and 2024	[32]
Romania, Timișoara, paediatric	2019 vs. 2021 vs. 2023	Carbapenem-resistant organisms peaked during the pandemic year at 8.81%; ESBL declined to 14.45% by 2023	[82]
Romania, south-east, 10 hospitals	Mar 2020–Mar 2022 vs. Apr 2022–Dec 2024	3929 HAI patients; longer stays during the pandemic; different comorbidity profile post-pandemic	[81]
Romania, Bucharest, congress report (Romanian)	2018 vs. 2022	CRE proportion among *K. pneumoniae* 27.6% to 34.8%; colistin resistance 52% among carbapenemase producers	[23]
Bulgaria, Plovdiv, urinary isolates	2017–2022, interrupted time series	ESBL production and carbapenem resistance both rose post-COVID; vancomycin resistance 4.6% to 8.9%	[87]
Greece, tertiary hospital bloodstream	2019–2025	KPC producers rose to 2022 then fell sharply; dual-carbapenemase and single-MBL producers rose steadily; colistin resistance rose continuously	[52]
Greece, Athens, CPE isolates	2020 vs. 2021 vs. 2022 vs. 2023	Resistance rose significantly in 2022–2023 vs. 2020 for nearly all agents including last-resort; rapid VIM to NDM transition	[50]
Greece, Thessaloniki, respiratory	2018–2023, three periods	Positive tracheal cultures 12.3% pre-pandemic, 21.9% peak in 2021, 18.3% in 2023	[84]
Greece, three tertiary hospitals	2019 vs. 2020–2022	Significant increases in CRE, CRAB and VRE in non-ICU wards during the pandemic	[85]
Croatia, national outpatient prescribing	2015–2024	Prescribing fell 21.0% in 2020 and returned to pre-pandemic levels by 2024	[88]
Croatia, three centres plus outpatient	Post-pandemic	Emergence of isolates with multiple carbapenemases; only 50% ceftazidime-avibactam susceptible, all imipenem-relebactam resistant	[62]
Slovenia, secondary-care hospital	Seven years, interrupted time series	MDR burden rose 4.93 to 5.81 per 1000 patient-days while MDR infections fell 1.61 to 1.29	[14]
Slovenia, national invasive isolates	2021 vs. 2022	Carbapenem resistance 0.9% to 1.9%; carbapenemase mix broadened from OXA-48-only to four enzymes	[19,20]
Serbia, Belgrade university hospital	Apr 2019–Mar 2020 vs. Apr 2020–Mar 2021 vs. Apr 2021–Mar 2022	Increased consumption, isolation rate, and resistance of *K. pneumoniae*, *P. aeruginosa*, and *A. baumannii*	[91]
Serbia, blood cultures	2020–2023	*K. pneumoniae* prevalence approximately 11.6% in 2020 and 2021, rising above 30% in 2022	[92]
Bosnia and Herzegovina, Banja Luka	2015–2024, ten-year series	Significant increase in invasive isolates during COVID; resistance rose significantly to cephalosporins, fluoroquinolones, and carbapenems	[93]
Montenegro, national	2011–2022	*K. pneumoniae* carbapenem resistance 17% (4/23) in 2019 to 47% (18/38) in 2022; consumption +14.04% in 2021 vs. 2020	[73]
North Macedonia, ICU	2020 vs. 2021	Total antibiotic use fell; carbapenem consumption rose from 1.7% to 14.37% of total	[94]
Slovakia, military hospital (regional comparator)	2017–2019 vs. 2020–2022	Relative CRKP incidence rose 4.8-fold, from 0.18% to 0.76%; 47% of CRKP patients also had COVID-19	[111]

CPE, carbapenemase-producing *Enterobacterales*; CRAB, carbapenem-resistant *Acinetobacter baumannii*; CRE, carbapenem-resistant *Enterobacterales*; ESBL, extended-spectrum β-lactamase; MBL, metallo-β-lactamase; MDR, multidrug-resistant; VRE, vancomycin-resistant enterococci.

**Table 6:** Clinical outcomes reported for carbapenem-resistant *Klebsiella pneumoniae* and related multidrug-resistant infections, Romania and South-Eastern Europe, 2020–2025.

Country, Setting	Population	Outcome Measure	Value (Ref.)
Romania, Bucharest	89 CRKP isolates	ICU stay → risk of sepsis/in-hospital death	10-fold/40.8-fold higher [95]
Romania	22-month cohort	Predictors of poor outcome (Cox HR): ICU admission/ward transitions/NDM type	HR 2.38/4.69/5.98 [96]
Romania, county emergency hospital	MDR HAIs, ~60% ICU-origin	Mortality	51.79% [118]
Greece, 3 hospitals	4370 inpatients	Rectal colonisation → same-species BSI	31.1% colonised; RR 5.2 (95% CI 3.9–6.8) [119]
Bulgaria	Haematology CRKP carriers	Progression to CRKP bacteraemia	21.4% [47]
Romania, Iași	CRE urinary tract infection cohort	ICU admission/case fatality, COVID+ vs. COVID−	66.7% vs. 18.2%/33.3% vs. 12.1% (*p* < 0.001) [79]
Romania	ICU COVID-19 cohort	Odds of death after superinfection	>15-fold increase [120]
Serbia	ICU, ventilated COVID-19, 64 MDR *A. baumannii* (clone IC2)	Mortality	100% [121]
Bulgaria	Neonatal ICU, VAP vs. no VAP	Length of stay/hospital cost	32 vs. 18 days (+14 d); €3675.77 vs. €2327.78 [122]

BSI, bloodstream infection; HR, hazard ratio; ICU, intensive care unit; MDR, multidrug-resistant; RR, relative risk; VAP, ventilator-associated pneumonia. No study in the region reports a CRKP-attributable mortality estimate against carbapenem-susceptible controls; values shown are crude outcome measures from the cited series, not adjusted attributable estimates.

**Table 7:** Reported in vitro susceptibility to novel β-lactam/β-lactamase-inhibitor combinations and cefiderocol among carbapenem-resistant *Klebsiella pneumoniae*, by country and carbapenemase profile, 2020–2025.

Country, Setting	Carbapenemase Profile	Agent	Susceptibility/Resistance (Ref.)
Greece, ICU	KPC-only	CAZ-AVI, MEV, IMI-REL	Susceptible, all three [53]
Serbia, Niš	OXA-48/KPC	CAZ-AVI	Highly active [68]
Serbia, Niš	Carbapenemase producers (mixed)	Cefiderocol	100% active [68]
Romania	NDM + OXA-48 co-producers	CAZ-AVI	83.7–100% resistant [97,98]
Romania	NDM + OXA-48 co-producers	Imipenem-relebactam	90.0% resistant [98]
Romania	NDM + OXA-48 co-producers	Ceftolozane-tazobactam	98.4% resistant [98]
Romania	NDM + OXA-48 co-producers	Aztreonam (alone)	98.4% resistant [98]
Croatia	Multiple carbapenemases (post-pandemic)	Imipenem-relebactam	100% resistant [62]
Croatia	Multiple carbapenemases (post-pandemic)	CAZ-AVI	50% susceptible [62]
Greece	Dual KPC-NDM	CAZ-AVI, MEV, IMI-REL	Resistant, all three [53]
Romania, 1-year survey	Carbapenemase producers (mixed)	Aztreonam + CAZ-AVI (synergy)	98.5% synergistic, 127/129 [98]
Romania, clinical series (*n* = 7)	NDM + OXA-48	Aztreonam + CAZ-AVI	Synergy 7/7; clinical recovery 7/7 [123]
Romania, ST383 WGS series	NDM-5 + OXA-48	Aztreonam-avibactam (fixed)	100% susceptible [77]
Romania, ST383 WGS series	NDM-5 + OXA-48	Cefiderocol (broth microdilution)	90.3% susceptible [77]
Romania, other centre	NDM + OXA-48	Cefiderocol	58.5% susceptible [32]
Romania, 1-year survey	Carbapenemase producers (mixed)	Cefiderocol	64.3% susceptible (35.7% resistant) [98]
Romania, 2-year series	Carbapenem-non-susceptible (mixed)	Cefiderocol	67.95% susceptible (32.05% resistant) [34]
Bulgaria	Carbapenemase producers (mixed)	Cefiderocol	37.5% (EUCAST) vs. 71.8% (CLSI) susceptible [101]
Croatia	Mixed	Cefiderocol	31 resistant isolates identified [63]
Greece, ST323 outbreak	KPC-2 + VEB-25	Cefiderocol	MIC 32 mg/L, resistant [56]

CAZ-AVI, ceftazidime-avibactam; IMI-REL, imipenem-relebactam; MEV, meropenem-vaborbactam; MIC, minimum inhibitory concentration; WGS, whole-genome sequencing. Breakpoint standard (EUCAST vs. CLSI) is not stated for all series; where both are reported (Bulgaria) both are shown, since they diverge materially.

## Data Availability

No new data were created or analyzed in this study. Data sharing is not applicable to this article.
